# Impact of varying levels of hyperglycemia on clinicoradiographic outcomes after endovascular reperfusion treatment

**DOI:** 10.1038/s41598-018-28175-6

**Published:** 2018-06-29

**Authors:** Seong-Joon Lee, Yang-Ha Hwang, Ji Man Hong, Jin Wook Choi, Bok Seon Yoon, Dong-Hun Kang, Yong-Won Kim, Yong-Sun Kim, Jeong-Ho Hong, Joonsang Yoo, Chang-Hyun Kim, Bruce Ovbiagele, Andrew M. Demchuk, Sung-Il Sohn, Jin Soo Lee

**Affiliations:** 1 Department of Neurology, Ajou University School of Medicine, Ajou University Medical Center, Suwon, South Korea; 2 Department of Neurology, Kyungpook National University School of Medicine and Hospital, Daegu, South Korea; 3 Department of Radiology, Ajou University School of Medicine, Ajou University Medical Center, Suwon, South Korea; 4 Department of Biomedical Sciences, Ajou University Graduate School of Medicine, Suwon, South Korea; 5 Department of Neurosurgery, Kyungpook National University School of Medicine and Hospital, Daegu, South Korea; 6 Department of Radiology, Kyungpook National University School of Medicine and Hospital, Daegu, South Korea; 7 Department of Neurology, Keimyung University Dongsan Medical Center, Daegu, Republic of Korea; 8 Department of Neurosurgery, Keimyung University Dongsan Medical Center, Daegu, Republic of Korea; 9 Department of Neurology, Medical University of South Carolina, Charleston, South Carolina USA; 10 Department of Clinical Neurosciences and Radiology, Hotchkiss Brain Institute, University of Calgary, Alberta, Canada

## Abstract

We evaluated the effects of admission hyperglycemia with different cut-off levels on 3-month outcomes, infarct growth, and hemorrhagic transformation in acute stroke patients with large artery occlusion of anterior circulation who received endovascular treatment (EVT). Between January 2011 and May 2016, patients that underwent EVT with pre-procedural and post-procedural diffusion-weighted imaging were identified from a multicenter registry. Normoglycemia was defined as a glucose level ≤ 110 mg/dL, moderate hyperglycemia as >110 and ≤170 mg/dL, and overt hyperglycemia as >170 mg/dL. Its effects on poor outcomes (3-month modified Rankin Scale score 3–6), infarct growth, and parenchymal hematoma type 2 were analyzed. Of 720 patients encountered, 341 patients were eligible. There was a statistically significant difference in glycated hemoglobin levels between the normoglycemia/moderate hyperglycemia and overt hyperglycemia groups (p < 0.001). Moderate hyperglycemia (odds ratio 2.37 [95% confidence interval 1.26–4.45], p = 0.007) and overt hyperglycemia (2.84 [1.19–6.81], p = 0.019) were associated with poor outcomes. Post-procedural infarct volumes were significantly greater in hyperglycemic patients (p_adjusted_ = 0.003). Only overt hyperglycemia (9.28 [1.66–51.88], p = 0.011) was associated with parenchymal hematoma type 2. Overall hyperglycemia was associated with poor outcomes and infarct growth, whereas overt hyperglycemia was associated with parenchymal hematoma type 2.

## Introduction

Presenting hyperglycemia and diabetes mellitus (DM) are predictors of a poor outcome after intravenous (IV) thrombolysis in patients with stroke^1^. They are also independently associated with early progression of stroke after thrombolysis^2^ as well as an increased risk of intracerebral hemorrhage (ICH)^3^. The mechanisms underlying these associations have been investigated mainly in rodent models of ischemia where reperfusion is guaranteed^4^. In contrast, reperfusion status has not been well validated in previous clinical studies of IV thrombolysis. This discrepancy has limited the generalizability of laboratory research findings to clinical research settings. Recently, major randomized controlled trials using new revascularization devices have established endovascular treatment (EVT) as the standard therapy for patients presenting with intracranial large vessel occlusion causing acute ischemic stroke^5–9^. Reported reperfusion rates after EVT range from 59% to 88%^5–9^, readdressing the classic ischemia/reperfusion model in real clinical settings.

Another obstacle to discriminating the effects of hyperglycemia is the difficulty in defining the respective effects of hyperglycemia and DM on patient outcomes. Additionally, stress hyperglycemia has been reported in patients with severe stroke or cardiac disease, even in the absence of DM. Although the classifications of normoglycemia, moderate (or non-diabetic) hyperglycemia, and overt (or diabetic) hyperglycemia vary in the literature, we have made an effort to approximate the most appropriate cut-off values using the ASIAN KR registry, which enrolled patients with acute ischemic stroke who received emergent EVT with modern devices and techniques at three Korean comprehensive stroke centers.

We hypothesized that moderate and overt hyperglycemia would exclusively affect clinical outcomes, infarct growth, and hemorrhagic transformation in patients with acute large artery occlusion and a high probability of successful reperfusion. To examine this hypothesis, we classified patients into normoglycemic, moderately hyperglycemic, and overtly hyperglycemic groups, and evaluated the effects of admission glucose level on clinical outcomes, infarct growth on diffusion-weighted imaging (DWI), and development of post-procedural parenchymal hematoma type 2 in the acute setting.

## Materials and Methods

The datasets generated during and/or analyzed during the current study are available from the corresponding author on reasonable request.

### Patient enrollment

The ASIAN KR registry, which includes data on 720 patients, was assembled for an observational study of consecutive patients aged 18 years or older who received EVT for the treatment of acute ischemic stroke caused by intracranial and/or extracranial large vessel occlusion^10^. The consecutive patient data were obtained from three comprehensive stroke centers in Korea (Ajou University Hospital [center A, Suwon], Kyungpook National University Hospital [center B, Daegu], and Keimyung University Dongsan Hospital [center C, Daegu]). De-identification and allocation of study identification numbers was undertaken for all clinical data. The data collection protocol was approved by the Institutional Review Board of each participating hospital and implemented in accordance with the ethical standards of the 1964 Declaration of Helsinki and its later amendments. The need for written informed consent was waived in view of the retrospective nature of the study.

To evaluate the effect of glucose level on the outcome of EVT, we applied the following inclusion criteria: (1) acute intracranial large artery occlusion in the anterior circulation; (2) onset-to-puncture time < 720 min, and (3) availability of both pre-procedural and post-procedural DWI volumes within 1 week of stroke onset. Pre-procedural brain computed tomography (CT) or magnetic resonance imaging (MRI) with collection of angiographic data was performed upon admission for each patient. Post-procedural CT or MRI of the brain was usually performed within 5 to 7 days of admission.

### Evaluations

Premorbid modified Rankin Scale (mRS) scores, National Institutes of Health Stroke Scale (NIHSS) scores on admission, and mRS scores at 3 months were analyzed. A 3-month mRS score of 0–2 or no change compared with the premorbid mRS score was classified as a good outcome and a 3-month mRS score of 3–6 was classified as a poor outcome. Routine laboratory results were also collected. After de-identification and blinding of the clinical data, stroke neurologists, neuroradiologists, and neurointerventionists with expertise in acute stroke management performed core laboratory imaging analyses to ensure consistent grading and eliminate possible bias. The location of the initial large vessel occlusion was identified on baseline angiography (SJL). Internal carotid artery (ICA) T, ICA I, middle cerebral artery (MCA) M1, and MCA M2 superior and inferior divisions were included in this study. ICA T was defined as an occlusive lesion in both M1 and ICA including or excluding the A1 segments. ICA I was defined as an occlusive intracranial lesion of the ICA sparing M1 and A1^11^. Alberta Stroke Program Early CT scores (ASPECTS) were classified on non-contrast CT (SIS). Successful reperfusion was defined as modified Treatment In Cerebral Ischemia (mTICI) grade 2b–3 (JSL, YHH)^12^. Post-procedural hemorrhagic complications were classified in accordance with the criteria defined by the European Cooperative Acute Stroke Study^13^. Subarachnoid hemorrhage (SAH) was classified in accordance with the modified Fisher scale (SIS)^14^. Parenchymal hematoma type 2 and/or grade 3–4 subarachnoid hemorrhage were regarded as serious post-procedural hemorrhagic complications. Pre-procedural and post-procedural DWI stroke volumes were evaluated (by JWC) using NordicICE semi-automated software (NordicNeuroLab, Bergen, Norway).

### Protocol and Procedures

The study protocols in participated centers were previously reported elsewhere^10^. Patients who presented to centers A and B between January 2011 and February 2016, and patients who visited center C between January 2011 and May 2016 were included. All centers used CT and CT angiography for baseline screening. IV tissue plasminogen activator (tPA) was given to patients that presented within 3 to 4.5 hours of onset and were indicated. If a large artery occlusion corresponding to the stroke signs was observed on CT angiography with an expected onset to puncture time <6 hours, EVT was considered in all patients if there were no contraindications. Patients with large core volumes were excluded according to protocols of each hospital. Center A excluded patients with low ASPECTS on non-contrast CT, and DWI were also utilized to rule out large infarct core volume. Center B excluded patients with ischemic lesions in non-contrast CT involving over half of the corresponding territory, or well developed hyperintensities on fluid attenuated inversion recovery imaging. Center C excluded patients with ASPECTS 0 ~3 on CT, and routinely utilized multiphase CT angiography since Aug. 2014 to rule out poor collaterals. When onset to puncture time was expected to be longer than 6 hours or onset time was unclear, further imaging modalities to select appropriate candidates were performed using multimodal MRI in the three hospitals.

The type of EVT procedure was chosen at the discretion of the treating physician. Direct aspiration and stent retrieval were primarily used in most cases^10,15–18^. Balloon guide catheters, intracranial or extracranial angioplasty, and/or stenting were implemented as needed.

### Grouping of presenting hyperglycemia and definition of DM

Blood glucose was routinely measured at admission, and glycated hemoglobin (HbA_1C_) was measured according to each respective stroke center’s protocol. Glucose status on admission was trichotomized into normoglycemia (≤110 mg/dL)^19^, moderate hyperglycemia (>110 mg/dL and ≤170 mg/dL), or overt hyperglycemia (>170 mg/dL)^20^, as evaluated by literature reviews and our preliminary analyses (Supplementary Table 1). Comorbid DM was defined as a previous history or diagnosis of DM, or an HbA1c on admission of >6.5%.

### Statistical analysis

Comparative analyses of the admission glucose level groups were performed for clinical characteristics, imaging findings, and treatment outcomes. Differences between the three groups were analyzed using the χ^2^ test for categorical variables or analysis of variance for continuous variables. To evaluate the effect of admission glucose level on patient outcome, we performed a logistic regression analysis that was adjusted for age, sex, premorbid mRS score, initial NIHSS score, IV tPA treatment, pre-procedural DWI volume, onset-to-puncture time, site of occlusion, final successful reperfusion, and serious post-procedural hemorrhagic complications as potential confounders. Infarct growth was evaluated in two ways. First, infarct growth (post-procedure DWI volume – pre-procedure DWI volume, mL) was compared between the groups. Second, to evaluate the effect of hyperglycemia on post-procedural infarct volume while controlling for baseline infarct volume, an analysis of covariance was performed comparing post-procedural DWI volumes between normoglycemic and hyperglycemic patients with a cut-off value of >110 mg/dL, adjusted for age, sex, pre-procedural DWI volume, site of occlusion, and final successful reperfusion. Finally, to evaluate the association between glucose status on admission and hemorrhagic complications, a logistic regression analysis was performed to assess the occurrence of parenchymal hematoma type 2 adjusted for age, sex, initial NIHSS score, IV tPA treatment, pre-procedure DWI volume, onset-to-puncture time, and site of occlusion. For subgroup analyses, the variables were evaluated again in the reperfusion (successful) group (post-procedural mTICI 2b – 3) and non-reperfusion (unsuccessful) group (mTICI 0–2a) for association between glucose levels and treatment outcomes, post-procedural infarct volume and occurrence of parenchymal hematoma type 2, respectively. The data are presented as the mean ± standard deviation or as the median (interquartile range). A p-value less than 0.05 was considered to be statistically significant. All statistical analyses were performed using IBM SPSS Statistics version 22 software (IBM Corp., Armonk, NY, USA).

## Results

### Baseline characteristics stratified by admission glucose status

Of the 720 patients in the registry, 341 met the inclusion criteria for this study. One hundred and ten of these patients presented with normoglycemia, 180 presented with moderate hyperglycemia, and 51 presented with overt hyperglycemia. The clinical characteristics, pre-procedural factors, reperfusion treatment, and outcomes according to glucose status on admission are outlined in Table 1. There was a statistically significant difference in glucose levels across the trichotomized groups by definition, but the presence of comorbid DM was significantly increased in the group with overt hyperglycemia (10.0% and 18.9% vs. 86.3%, respectively; p < 0.001). Similar trends were observed for HbA_1c_ levels (5.7% ± 0.5% and 5.9% ± 0.6% vs. 8.1% ± 1.9%, respectively; p < 0.001). Initial NIHSS scores were not significantly different between the groups (15.0 [11.00–18.25] vs. 16.0 [12.0–20.0] vs. 16.0 [13.0–20.0], respectively; p = 0.084). In terms of laboratory data, white blood cell count, and erythrocyte sedimentation rate on admission increased significantly in hyperglycemia groups.

**Table 1:** Baseline Characteristics and Pre-procedural Factors of Included Patients Grouped According to Glucose Level on Admission.

	Normoglycemia (n = 110)	Moderate hyperglycemia (n = 180)	Overt hyperglycemia (n = 51)	p-value
**Clinical characteristics**				
Age, years	65.8 ± 14.5	68.5 ± 11.4	66.5 ± 11.7	0.190
Male sex	67 (60.9%)	99 (55.0%)	27 (52.9%)	0.523
Hypertension	61 (55.5%)	113 (62.8%)	33 (64.7%)	0.380
Diabetes mellitus	11 (10.0%)	34 (18.9%)	44 (86.3%)	<0.001
Atrial fibrillation	49 (44.5%)	101 (56.1%)	23 (45.1%)	0.110
Smoking	29 (26.4%)	42 (23.3%)	13 (25.5%)	0.835
Premorbid mRS	0.0 [0.0–0.0]	0.0 [0.0–0.0]	0.0 [0.0–0.0]	0.445
Admission NIHSS score	15.0 [11.00–18.25]	16.0 [12.0–20.0]	16.0 [13.0–20.0]	0.084
**Laboratory data**				
Glucose (mg/dL)	98.6 ± 9.1	130.3 ± 14.6	236.2 ± 61.9	<0.001*
HbA1c (%)	5.7 ± 0.5	5.9 ± 0.6	8.1 ± 1.9	<0.001^†^
Hemoglobin	13.4 ± 1.9	13.6 ± 1.7	13.4 ± 2.0	0.761
WBC	7.8 ± 2.3	8.6 ± 3.0	8.1 ± 2.8	0.038^‡^
Platelet	223.4 ± 70.8	223.2 ± 77.0	225.4 ± 60.3	0.983
ESR (mg/dL)	12.4 ± 11.8	12.8 ± 13.4	20.2 ± 18.3	0.001*
**Pre-procedural imaging factors**				
Intracranial occlusion				0.182
ICA T	36 (32.7%)	50 (27.8%)	8 (15.7%)	
ICA I	7 (6.4%)	12 (6.7%)	3 (5.9%)	
MCA M1	55 (50.0%)	102 (56.7%)	36 (70.6%)	
MCA M2 superior	5 (4.5%)	10 (5.6%)	0 (0.0%)	
MCA M2 inferior	7 (6.4%)	6 (3.3%)	4 (7.8%)	
ASPECTS	8.0 [5.0–9.0]	7.0 [5.0–9.0]	7.0 [5.0–9.0]	0.892
Pre-procedural DWI volume (mL)	23.5 ± 29.1	23.0 ± 30.9	23.5 ± 33.9	0.990
Onset to pre-procedural MRI (min)	225 ± 159	234 ± 143	247 ± 171	0.694

The data are presented as the mean ± standard deviation, number (%), or median [interquartile range]. *Normoglycemia vs. moderate hyperglycemia vs. overt hyperglycemia, p < 0.05, Bonferroni post-hoc test; ^†^normoglycemia/moderate hyperglycemia vs. overt hyperglycemia, p < 0.05, Bonferroni post-hoc test; ^‡^normoglycemia vs. moderate hyperglycemia, p < 0.05, Bonferroni post-hoc test. HbA_1c_, glycated hemoglobin; mRS, modified Rankin Scale; IQR, interquartile range; NIHSS, National Institutes of Health Stroke Scale; SBP, systolic blood pressure; DBP, diastolic blood pressure; MBP, mean blood pressure; WBC, white blood cell; ESR, erythrocyte sedimentation rate; ICA, internal carotid artery; MCA, middle cerebral artery; ASPECTS, Alberta Stroke Program Early CT score; DWI, diffusion-weighted imaging.

### Reperfusion treatment and outcomes

In the pre-procedural imaging data, the initial site of occlusion, ASPECTS, and pre-procedural DWI stroke volumes did not differ significantly between the three groups. Onset to pre-procedural MRI, onset-to-puncture time, procedure time, and onset to post-procedural MRI also did not differ significantly between the three groups.

In terms of reperfusion methods and outcomes (Table 2), treatment with IV tPA was more common in the normoglycemia and moderate hyperglycemia groups than in the overt hyperglycemia group (66.4% and 60.6% vs. 39.2%, respectively; p = 0.004). Regarding EVT, there was no significant difference in use of stent retrieval and direct aspiration methods between the three groups. There was also no significant difference in the rate of achievement of successful reperfusion between the three groups.

**Table 2:** Reperfusion Treatment and Outcomes According to Glucose Level Groups.

	Normoglycemia (n = 110)	Moderate hyperglycemia (n = 180)	Overt hyperglycemia (n = 51)	p-value
**Reperfusion treatment**				
IV tPA infusion	73 (66.4%)	109 (60.6%)	20 (39.2%)	0.004
Onset-to-puncture time (min)	286 ± 165	298 ± 145	307 ± 173	0.680
Procedure time (min)	71.1 ± 39.2	69.9 ± 40.0	67.5 ± 45.9	0.874
Successful reperfusion	84 (76.4%)	146 (81.1%)	42 (82.4%)	0.548
**Outcomes**				
Hemorrhagic transformation				0.045
No hemorrhage	86 (78.2%)	132 (73.3%)	30 (58.8%)	
HI type 1	11 (10.0%)	15 (8.3%)	3 (5.9%)	
HI type 2	4 (3.6%)	18 (10.0%)	8 (15.7%)	
PH type 1	6 (5.5%)	10 (5.6%)	5 (9.8%)	
PH type 2	3 (2.7%)	5 (2.8%)	5 (9.8%)	
Post-procedural DWI volume (mL)	49.1 ± 53.5	64.0 ± 77.8	71.5 ± 93.5	0.126
Onset to post-procedural MRI (min)	6109 ± 2462	6218 ± 2478	5865 ± 2404	0.662
Infarct growth (mL)	25.6 ± 40.8	41.0 ± 65.3	48.0 ± 79.4	0.045^*^
3-month mRS 3–6	28 (25.5%)	76 (42.2%)	23 (45.1%)	0.007

The data are presented as the mean ± standard deviation, or number (%). *Normoglycemia vs. moderate hyperglycemia, p = 0.114; normoglycemia vs. overt hyperglycemia, p = 0.095, Bonferroni post-hoc test. IV tPA, intravenous tissue plasminogen activator; DWI, diffusion-weighted imaging; HI, hemorrhagic infarct; PH, parenchymal hematoma.

Poor outcomes at 3 months weres significantly more common in the moderate and overt hyperglycemia groups than in the normoglycemia group (42.2% and 45.1% vs. 25.5%, respectively; p = 0.007). When glucose status on admission was incorporated into a logistic regression model for poor outcome (Table 3), both moderate hyperglycemia (odds ratio [OR] 2.37, 95% confidence interval [CI] 1.26–4.45, p = 0.007) and overt hyperglycemia (OR 2.84, 95% CI 1.19–6.81, p = 0.019) were independent predictors of a poor outcome when compared with normoglycemia as a reference, suggesting a cut-off admission glucose value of >110 mg/dL for a poor prognosis. When logistic regression analysis was performed on the basis of reperfusion status, this association was still seen in the non-reperfusion group (n = 69) but not in the reperfusion group (n = 272).

**Table 3:** Logistic Regression Model of Hyperglycemia as a Risk Factor for a Poor 3-Month Outcome in Patients with Acute Ischemic Stroke of the Anterior Circulation after Endovascular Revascularization Therapy According to Reperfusion Status.

	Overall (n = 341)		Reperfusion subgroup (n = 272)		Non-reperfusion subgroup (n = 69)	
	OR (95% CI)	p-value	OR (95% CI)	p-value	OR (95% CI)	p-value
Age	1.05 (1.03–1.08)	<0.001	1.05 (1.02–1.08)	0.001	1.07 (1.01–1.14)	0.030
Sex	0.94 (0.55–1.62)	0.826	0.92 (0.49–1.71)	0.793	1.43 (0.36–5.73)	0.616
Premorbid mRS score		0.083		0.126		0.598
mRS0	Reference		Reference		Reference	
mRS1	2.37 (1.05–5.35)	0.039	2.40 (0.94–6.14)	0.068	4.72 (0.50–44.61)	0.176
mRS2	4.53 (1.13–18.18)	0.033	4.69 (1.08–20.44)	0.040	—	0.999
mRS3	1.13 (0.23–5.58)	0.879	1.81 (0.29–11.21)	0.525	0.47 (0.01–21.22)	0.700
mRS4	2.12 (0.06–78.41)	0.684	2.43 (0.07–89.64)	0.629		
Admission NIHSS score	1.12 (1.06–1.19)	< 0.001	1.12 (1.05–1.20)	0.001	1.19 (1.01–1.39)	0.035
IV tPA	1.134 (0.58–2.22)	0.714	1.14 (0.52–2.50)	0.735	1.49 (0.22–10.15)	0.682
Baseline intracranial occlusion		0.917		0.775		0.904
ICA T	Reference		Reference		Reference	
ICA I	1.01 (0.33–3.09)	0.993	0.88 (0.27–2.95)	0.840	3.68 (0.03–503.34)	0.604
MCA M1	1.00 (0.54–1.88)	0.990	0.92 (0.45–1.87)	0.822	0.84 (0.16–4.46)	0.835
MCA M2 superior	0.54 (0.12–2.32)	0.403	0.29 (0.05–1.94)	0.203	1.20 (0.22–6.44)	0.999
MCA M2 inferior	1.28 (0.36–4.62)	0.706	1.21 (0.27–5.44)	0.804	—	0.471
Pre-treatment DWI volume	1.02 (1.01–1.03)	0.002	1.02 (1.01–1.03)	0.002	1.00 (0.98–1.02)	0.704
Onset to puncture	1.00 (1.00–1.00)	0.044	1.00 (1.00–1.01)	0.025	1.00 (1.00–1.01)	0.604
Final mTICI 2b–3	0.24 (0.12–0.46)	<0.001				
PH2 or SAH 3–4	8.25 (2.15–31.66)	0.002	3.97 (0.88–17.89)	0.073	—	0.999
Glucose level		0.014		0.171		0.007
Normoglycemia	Reference		Reference		Reference	
Moderate hyperglycemia	2.37 (1.26–4.45)	0.007	1.86 (0.90–3.82)	0.094	10.99 (2.15–56.25)	0.016
Overt hyperglycemia	2.84 (1.19–6.81)	0.019	2.24 (0.83–6.02)	0.112	35.5 (1.93–653.02)	0.002

OR, odds ratio; CI, confidence interval; mRS, modified Rankin Scale; NIHSS, National Institutes of Health Stroke Scale; IV tPA, intravenous tissue plasminogen activator; ICA, internal carotid artery; MCA, middle cerebral artery; DWI, diffusion-weighted imaging; mTICI, modified Treatment In Cerebral Ischemia; PH2, parenchymal hematoma type 2; SAH, subarachnoid hemorrhage.

### Infarct growth

**Figure 1:**
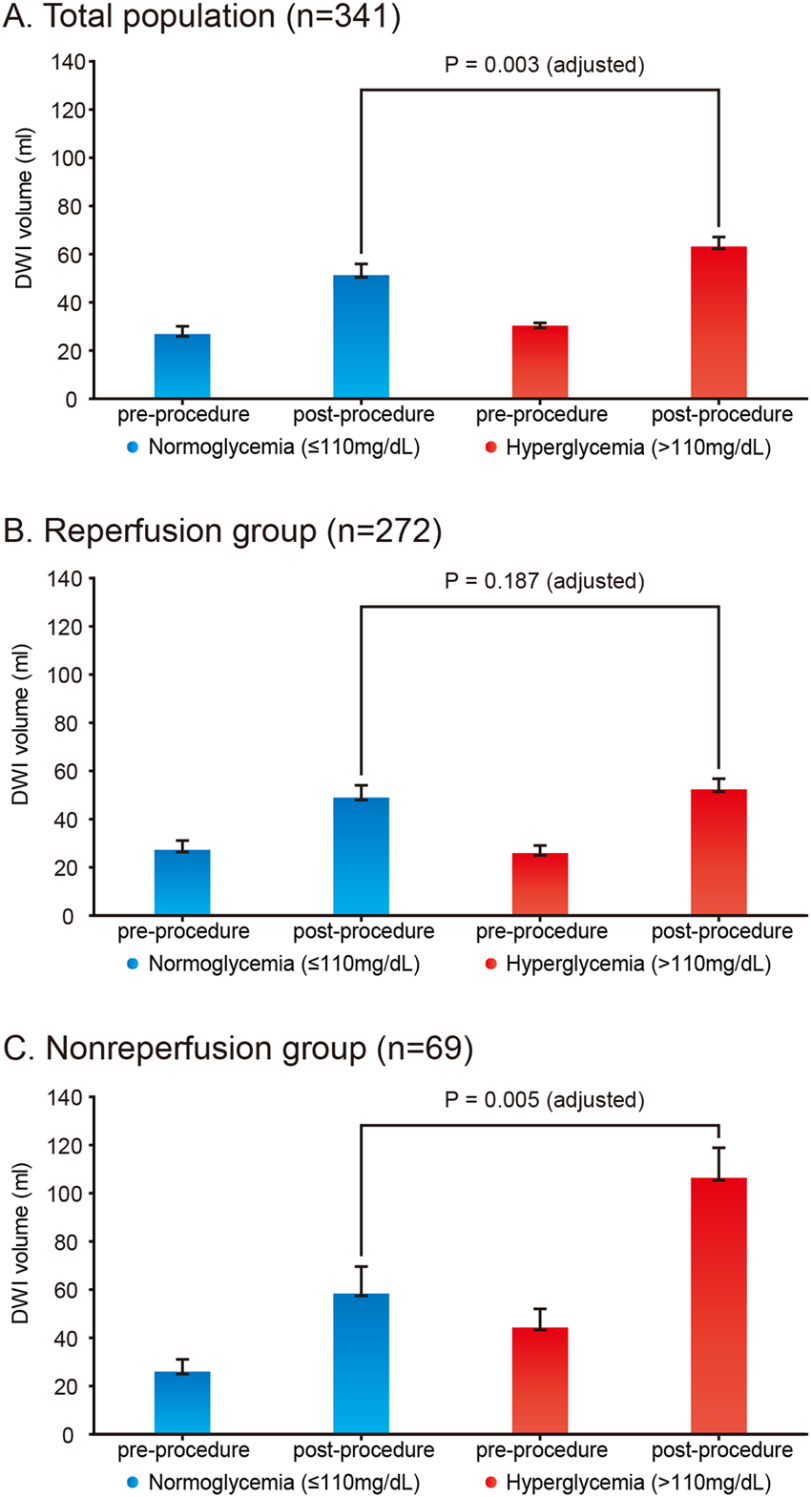
Changes in Pre-procedural and Post-procedural Diffusion-Weighted Image Volume According to the Presence of Hyperglycemia on Admission. (**A**) Total population, (**B**) reperfusion subgroup, and (**C**) non-reperfusion subgroup. Post-procedural infarct volumes were compared using analysis of covariance and adjusting for pre-procedural DWI volume, age, sex, site of occlusion, and successful reperfusion.; DWI, diffusion-weighted imaging.

When infarct growth was compared according to glucose status on admission, there was a significant intergroup difference (25.6 ± 40.8 mL vs. 41.0 ± 65.3 mL vs. 48.0 ± 79.4 mL, respectively; p = 0.045). When patients were dichotomized into normoglycemia and hyperglycemia groups (cut-off glucose level, 110 mg/dL), post-procedural DWI stroke volume was significantly greater in the hyperglycemia group (49.1 ± 53.5 mL vs. 65.6 ± 81.4 mL, respectively; p_adjusted_ = 0.003) although pre-procedural volumes were not significantly different (23.5 ± 29.1 mL vs. 23.1 ± 31.5 mL, p = 0.913). Similar to the clinical outcomes, this difference was significant in the non-reperfusion group (normoglycemia vs. hyperglycemia; 54.0 ± 58.2 mL vs. 126.0 ± 109.5 mL, p_adjusted_ = 0.005), but not for the reperfusion groups (normoglycemia vs. hyperglycemia; 47.6 ± 52.2 mL vs. 51.9 ± 66.5 mL, p_adjusted_ = 0.187, Fig. 1). Analyses of Clinical characteristics, pre-procedural factors, reperfusion treatment, and outcomes not shown in Table 1 are shown in Supplementary Table 2.

### Hemorrhagic complications

Hemorrhagic complications increased mainly in the overt hyperglycemia group (p = 0.045) (Table 2). When glucose status on admission was incorporated into a logistic regression model to assess hyperglycemia as a risk factor for parenchymal hematoma type 2 (Table 4), only overt hyperglycemia showed an independent association (OR 9.28, CI 1.66–51.88, p = 0.011) referenced by normoglycemia. In terms of post-procedural reperfusion status, overt hyperglycemia was associated with parenchymal hematoma type 2 only in the reperfusion group (OR 12.34, CI 1.60–95.07), as referenced to normoglycemia, p = 0.016), but the association was insignificant in the non-reperfusion group. With the same covariable adjustment, overt hyperglycemia was also confirmed to be as an independent predictor of parenchymal hematoma type 1–2 (OR 3.75, CI 1.25–11.23, p = 0.018) and any type of hemorrhagic transformation (OR 3.10, CI 1.39–6.92, p = 0.006).

**Table 4:** Logistic Regression Model of Hyperglycemia as a Risk Factor for Post-procedural Parenchymal Hematoma Type 2 in Patients with Acute Ischemic Stroke of the Anterior Circulation after Endovascular Revascularization Therapy According to Reperfusion Status.

	Overall (n = 341)		Reperfusion subgroup (n = 272)		Non-reperfusion subgroup (n = 69)	
	OR (95% CI)	p-value	OR (95% CI)	p-value	OR (95% CI)	p-value
Age	0.99 (0.94–1.04)	0.670	1.00 (0.93–1.04)	0.542	—	1.000
Sex	1.89 (0.51–6.98)	0.342	1.30 (0.33–5.08)	0.702	—	1.000
Admission NIHSS score	1.04 (0.92–1.17)	0.583	1.03 (0.90–1.19)	0.634	—	1.000
IV tPA	0.93 (0.21–4.11)	0.926	1.22 (0.24–6.33)	0.810	—	0.999
Baseline intracranial occlusion		0.048		0.111		1.000
ICA T	Reference		Reference		Reference	
ICA I	0.00 (0.00)	0.998	0.00 (0.00)	0.998	—	1.000
MCA M1	0.15 (0.03–0.66)	0.012	0.09 (0.02–0.52)	0.007	—	1.000
MCA M2 superior	3.66 (0.60–22.21)	0.159	1.42 (0.14–14.36)	0.768	—	0.999
MCA M2 inferior	0.00 (0.00)	0.998	0.00 (0.00)	0.999	—	1.000
Pre-procedure DWI volume	1.01 (1.00–1.03)	0.126	1.01 (1.00–1.03)	0.130	—	1.000
Onset to puncture	1.00 (1.00–1.01)	0.730	1.00 (1.00–1.01)	0.300	—	0.999
Final mTICI 2b–3	1.52 (0.28–8.14)	0.624				
Glucose level		0.009		0.025		1.000
Normoglycemia	Reference		Reference		Reference	
Moderate hyperglycemia	0.92 (0.20–4.36)	0.920	1.39 (0.24–8.15)	0.717	—	1.000
Overt hyperglycemia	9.28 (1.66–51.88)	0.011	12.34 (1.60–95.07)	0.016	—	1.000

OR, odds ratio; CI, confidence interval; NIHSS, National Institutes of Health Stroke Scale; IV tPA, intravenous tissue plasminogen activator; ICA, internal carotid artery; MCA, middle cerebral artery; DWI, diffusion-weighted imaging; mTICI, modified Treatment In Cerebral Ischemia.

## Discussion

The present study demonstrates that high glucose levels on admission are independently associated with poor 3-month outcomes, infarct growth, and significant hemorrhagic complications; however, the glucose level cut-off points for predicting these outcomes varied. Overall, hyperglycemia was associated with a poor functional outcome and infarct growth, which were predominantly seen in the non-reperfusion subgroup. In contrast, overt hyperglycemia was associated with hemorrhagic complications, which were predominant in the reperfusion subgroup. Ten percent of patients in the normoglycemia group had DM, 18% in the moderate hyperglycemia group, and 80% in the overt hyperglycemia group, with a similar trend observed for mean HbA_1c_ levels, suggesting that the overt hyperglycemia group could have had sustained hyperglycemia, while the moderate hyperglycemia group might be more representative of hyperglycemia under non-diabetic conditions.

The poor outcomes were in parallel with infarct growth in terms of the cut-off level of hyperglycemia on admission, which was relatively low. Post-procedural infarct volumes were significantly larger in the moderate and overt hyperglycemia groups than in the normoglycemia group, and both groups were independently associated with poor outcome. On this basis, infarct growth seems to be a mediator between hyperglycemia and a poor outcome; however, there was no correlation between glucose level on admission and infarct growth nor an interaction between glucose level and infarct growth in terms of poor outcomes (data not shown). This suggests that this association may not be linear, and an approach using cut-off values may better represent the phenomenon, as shown in our study. Regarding the association with poor outcomes or infarct growth, the significance of glucose levels as an independent predictor disappeared in multivariable analysis when only patients with successful reperfusion were analyzed, whereas both significant associations were still present in patients with unsuccessful reperfusion. This finding implies that successful reperfusion may overcome the negative effects of hyperglycemia. This observation is partly supported by a recent randomized controlled trial known as MR CLEAN, which showed no interaction of hyperglycemia and EVT effect when compared with non-endovascular treatment, indicating that hyperglycemia on admission is not a contraindication in candidates for EVT^21^.

Several mechanisms via which hyperglycemia leads to poor outcomes and infarct growth can be postulated, focusing on moderate hyperglycemia. First, the concept of stress hyperglycemia can be applied to our findings. A previous meta-analysis demonstrated a strong correlation between glucose levels > 110 to 126 mg/dL and poor outcomes only in non-diabetic patients with acute ischemic stroke, leading to this concept^19^. In the literature, glucose levels have been shown to increase with increasing stroke severity via activation of the hypothalamic-pituitary-adrenal axis^22,23^. Second, the possibility of a direct contribution of hyperglycemia itself to the aggravation of ischemic stroke needs to be considered. Infarct growth can be precipitated by decreased reperfusion and penumbral salvage, both of which are associated with hyperglycemia^4,24^. Several studies have reported the deleterious effects of hyperglycemia in patients with non-lacunar focal ischemia and global ischemia. Further, hyperglycemia has been associated with reduced penumbral salvage in patients with perfusion-diffusion mismatch^25,26^. A third mechanism for understanding the impact of moderate hyperglycemia on acute stroke that is worth discussing is that presenting hyperglycemia may represent pre-existing abnormalities in glucose metabolism. A large number of patients with ischemic stroke and no history of DM are found to have insulin resistance, impaired glucose metabolism, or DM at follow-up^27,28^. While presenting hyperglycemia does not represent overt DM, it may reflect insulin resistance and comprise the metabolic syndrome, which is known to be associated with poor leptomeningeal collateral status in acute ischemic stroke^29^. This could be an alternative but complementary explanation for predominant infarct growth in the non-reperfusion subgroup seen in our study. Pre-diabetic conditions were not addressed in our study, and further insights are needed to address this issue.

In the present study, overt hyperglycemia, which is more clearly indicative of diabetic comorbidity, was associated with severe intracerebral hemorrhagic complications, especially in the reperfusion subgroup. Our findings suggest that reperfusion injury can be exacerbated by chronic sustained hyperglycemia. The association between DM and ICH in patients with ischemic stroke, especially after IV thrombolysis, is well recognized^30^. However, IV rt-PA was not a predictor of parenchymal hematoma and was not associated with the clinical outcomes in our endovascular population. An association between sustained hyperglycemia or DM and hemorrhagic transformation has been recently reported in endovascular populations; however, in one study, the endovascular devices and methods used were somewhat outdated^31^, and in another study using stent retrievers, the significance for hyperglycemia was not shown, but DM was confirmed to be significant^32^. Exacerbated reperfusion injury in overt hyperglycemia may be explained as follows. Oxidative stress and activation of inflammation are reported to be aggravated, resulting in dysfunction of the blood-brain barrier^33–35^. Moreover, severe hyperglycemia significantly worsens cortical intracellular acidosis in the brain and mitochondrial dysfunction in the ischemic penumbra^36^. Such mechanisms can lead to increases in hemorrhagic transformation and extensive hemorrhage, as revealed in a feline model of MCA occlusion^37^. An elevated erythrocyte sedimentation rate, which indicates an inflammatory reaction, was seen in patients with overt hyperglycemia in our study.

A previous large-scale study of patients who received IV tPA yielded results similar to those of the present study in terms of glucose cut-off values. The Safe Implementation of Treatment in Stroke International Stroke Thrombolysis Register (SITS-ISTR), a study involving over 16,000 patients with acute ischemic stroke who were treated with thrombolysis, showed that glucose levels > 120 mg/dL were associated with increased mortality, while levels > 180 mg/dL were associated with symptomatic ICH per the SITS-MOST criteria^38^. In a subgroup analysis according to history of diabetes, the ORs for mortality and functional dependence were significantly higher for glucose levels > 120 mg/dL in non-diabetic patients, while the values for mortality and functional independence were 181–200 mg/dL and 160 mg/dL, respectively, in diabetic patients. The OR for symptomatic ICH per the SITS-MOST criteria was significantly higher in patients with glucose levels of 181–200 mg/dL compared with that for patients who had lower glucose levels with or without a history of diabetes. The SITS-ISTR results and our present findings both provide dual glucose cut-off values at similar ranges, with the lower value showing an association with poor outcome and the higher value showing an association with hemorrhagic complications. Our present research also reveals that higher glucose cut-off values may be associated with sustained hyperglycemia and comorbidity of DM.

The present study has some limitations. First, although our analysis included multicenter data, it was limited by an observational study design. Nonetheless, the population was medium-sized and presented with acceptable revascularization profiles. Second, the retrospective study design precluded the use of symptomatic ICH as an endpoint, which is widely used in EVT trials. However, parenchymal hematoma type 2, which was used in the current study, is known to be strongly associated with neurologic deterioration^39^. Third, patients were only included when both pre-procedural and post-procedural MRI data were available, allowing for clinical evaluation of infarct growth. However, post-procedural MRI data were not available for patients with severe pathology or for those who died, such that clinical outcomes in our study population may have been positively overestimated. Nevertheless, the analysis used both pre-procedural and short-term post-procedural DWI, which is very rare, so we believe that this study must have a novel value. Finally, the management of hyperglycemia, which can subsequently affect imaging and clinical outcomes, might have differed between hospitals and attending physicians. Management of hyperglycemia is another unresolved issue in acute ischemic stroke that is currently being investigated in therapeutic trials, such as the Stroke Hyperglycemia Insulin Network Effort (SHINE) trial^40^.

In conclusion, moderate to overt hyperglycemia on admission was associated with a poor outcome and infarct growth in Korean patients with acute intracranial large artery occlusion who underwent EVT but did not achieve successful reperfusion. Additionally, overt hyperglycemia was associated with significant hemorrhagic complications, especially in patients with successful reperfusion. These findings should be confirmed in future large-scale prospective cohorts.

## Electronic supplementary material

Supplementary table 1 and 2
