# Supplementary material for: Impact of varying levels of hyperglycemia on clinicoradiographic outcomes after endovascular reperfusion treatment
Source: Sci Rep. 2018 Jun 29;8:9832. doi: 10.1038/s41598-018-28175-6 (PMC6026188; doi:10.1038/s41598-018-28175-6)
Supplement: Supplementary file 1 — Supplementary table 1 and 2 [file 41598_2018_28175_MOESM1_ESM.docx]

**Impact of varying levels of hyperglycemia on clinicoradiographic outcomes after endovascular reperfusion treatment**

**Running title:** Hyperglycemia and endovascular treatment

Seong-Joon Lee^1,¶^, Yang-Ha Hwang^2,¶^, Ji Man Hong^1^, Jin Wook Choi^3^, Bok Seon Yoon^4^, Dong-Hun Kang^5,6^, Yong-Won Kim^2,6^, Yong-Sun Kim^6^, Jeong-Ho Hong^7^, Joonsang Yoo^7^, Chang-Hyun Kim^8^, Bruce Ovbiagele^9^, Andrew M. Demchuk^10^, Sung-Il Sohn^7,^*, Jin Soo Lee^1,4^*

^1^ Department of Neurology, Ajou University School of Medicine, Ajou University Medical Center, Suwon, South Korea

^2^ Department of Neurology, Kyungpook National University School of Medicine and Hospital, Daegu, South Korea

^3^ Department of Radiology, Ajou University School of Medicine, Ajou University Medical Center, Suwon, South Korea

^4^ Department of Biomedical Sciences, Ajou University Graduate School of Medicine, Suwon, South Korea

^5^ Department of Neurosurgery, Kyungpook National University School of Medicine and Hospital, Daegu, South Korea

^6^ Department of Radiology, Kyungpook National University School of Medicine and Hospital, Daegu, South Korea

^7^ Department of Neurology, Keimyung University Dongsan Medical Center, Daegu, Republic of Korea

^8^ Department of Neurosurgery, Keimyung University Dongsan Medical Center, Daegu, Republic of Korea

^9^ Department of Neurology, Medical University of South Carolina, Charleston, South Carolina, USA

^10^ Department of Clinical Neurosciences and Radiology, Hotchkiss Brain Institute, University of Calgary, Alberta, Canada

* Corresponding authors

**Corresponding authors**

Jin Soo Lee, MD, PhD

Department of Neurology, Ajou University School of Medicine

San 5, Woncheon-dong, Yeongtong-gu, Suwon, Kyungki-do, 443–721, South Korea

Phone: +82-31-219-5175

Fax: +82-31-219-5178

E-mail: jinsoo22@gmail.com

Sung-Il Sohn M.D., Ph.D.

Department of Neurology, Dongsan Medical Center, Brain Research Institute, Keimyung University School of Medicine,

56 Dalseong-ro Joong-gu, Daegu, 41931, Republic of Korea

Tel : 82-53-250-7075

Fax : 82-53-250-7830

Email: sungil.sohn@gmail.com

^¶^Both authors contributed equally to this study.

**Supplementary Table 1.** Association between hyperglycemia and poor outcomes, parenchymal hematoma type 2, and increase in DWI lesion volume using different cut-off values to define hyperglycemia.

| Glucose cut-off value for hyperglycemia (mg/dL) | Poor outcomes* | | Parenchymal hematoma type 2^†^ | | Post-procedure diffusion weighted image volume^‡^ | | |
| --- | --- | --- | --- | --- | --- | --- | --- |
|  | OR | p-value | OR | p-value | Normoglycemia | Hyperglycemia | p-value |
| >80 | - | 0.928 | - | 0.999 | - | - | 0.758 |
| >90 | - | 0.421 | - | 0.998 | - | - | 0.814 |
| >100 | 3.17 (1.39–7.20) | 0.006 | - | 0.942 | - | - | 0.110 |
| >110 | 2.46 (1.33–4.53) | 0.004 | - | 0.433 | 49.1±53.5 | 65.7±81.4 | 0.003 |
| >120 | 2.36 (1.35–4.12) | 0.003 | 4.15 (1.04–16.57) | 0.044 | 53.7±65.1 | 66.6±81.0 | 0.004 |
| >130 | 1.76 (1.01–3.04) | 0.045 | - | 0.197 | 56.3±69.6 | 67.1±80.3 | 0.022 |
| >140 | - | 0.145 | - | 0.153 | 56.2±67.7 | 71.2±87.3 | 0.017 |
| >150 | - | 0.528 | 4.31 (1.17–15.88) | 0.028 | - | - | 0.343 |
| >160 | - | 0.314 | 5.83 (1.54–22.08) | 0.009 | - | - | 0.216 |
| >170 | - | 0.213 | 9.79 (2.30–41.62) | 0.002 | - | - | 0.093 |
| >180 | - | 0.121 | 11.60 (2.63–51.24) | 0.001 | - | - | 0.057 |
| >190 | - | 0.290 | - | 0.052 | - | - | 0.155 |
| >200 | - | 0.967 | 6.94 (1.42–33.96) | 0.017 | - | - | 0.673 |

*Adjusted by age, sex, premorbid modified Rankin Scale, admission National Institutes of Health Stroke Scale score, intravenous tissue plasminogen activator, baseline occlusion, baseline DWI volume, onset-to-puncture time, reperfusion, and presence of parenchymal hematoma type 2 or subarachnoid hemorrhage type 3–4.

^†^Adjusted by age, sex, National Institutes of Health Stroke Scale score on admission, intravenous tissue plasminogen activator, baseline occlusion, baseline DWI volume, onset-to-puncture time, and reperfusion.

^‡^Adjusted by age, sex, baseline occlusion, reperfusion, and initial DWI volume.

DWI, diffusion-weighted imaging; OR, odds ratio

**Supplementary Table 2.** Clinical characteristics, pre-procedural factors, reperfusion treatment, and outcomes not shown in Table 1 in enrolled patients according to presence of hyperglycemia.

|  | Normoglycemia (n=110) | Moderate hyperglycemia (n=180) | Overt hyperglycemia  (n=51) | p-value |
| --- | --- | --- | --- | --- |
| **Clinical characteristics** |  |  |  |  |
| Smoking | 29 (26.4%) | 42 (23.3%) | 13 (25.5%) | 0.835 |
| Atrial fibrillation | 49 (44.5%) | 101 (56.1%) | 23 (45.1%) | 0.110 |
| CAOD | 12 (10.9%) | 22 (12.2%) | 6 (11.8%) | 0.945 |
| Hypercholesterolemia | 29 (26.4%) | 57 (31.7%) | 21 (41.2%) | 0.168 |
| **Laboratory data** |  |  |  |  |
| SBP | 144.5±25.8 | 147.8±27.4 | 156.2±32.8 | 0.046* |
| DBP | 79.8±15.2 | 84.1±14.8 | 86.6±15.0 | 0.012* |
| MBP | 101.4 ±16.3 | 105.3±16.9 | 109.8±19.2 | 0.012* |
| Hemoglobin | 13.4±1.9 | 13.6±1.7 | 13.4±2.0 | 0.761 |
| WBC count | 7.8±2.3 | 8.6±3.0 | 8.1±2.8 | 0.038^†^ |
| Platelet count | 223.4±70.8 | 223.2 ±77.0 | 225.4±60.3 | 0.983 |
| ESR (mg/dL) | 12.4±11.8 | 12.8±13.4 | 20.2±18.3 | 0.001* |
| **MRI imaging time** |  |  |  |  |
| Onset to pre-procedural MRI (min) | 225±159 | 234±143 | 247±171 | 0.694 |
| Onset to post-procedural MRI (min) | 6109±2462 | 6218±2478 | 5865±2404 | 0.662 |
| **Reperfusion treatment** |  |  |  |  |
| Stent retrieval performed | 58 (52.7%) | 102 (56.7%) | 27 (52.9%) | 0.773 |
| Direct aspiration performed | 79 (71.8%) | 123 (68.3%) | 38 (74.5%) | 0.641 |
| SAH grade 3–4 | 3 (2.7%) | 1 (0.6%) | 0 (0.0%) | 0.175 |
| **Outcomes** |  |  |  |  |
| 7-day NIHSS score, median [IQR] | 3.0 [2.0–11.0] | 7.0 [2.25–13.75] | 6.5 [2.0–15.0] | 0.012^†^ |
| 3-month mRS |  |  |  | 0.005 |
| 0 | 17 (15.5%) | 35 (19.4%) | 10 (19.6%) |  |
| 1 | 41 (37.3%) | 35 (19.4%) | 8 (15.7%) |  |
| 2 | 22 (20.0%) | 31 (17.2%) | 9 (17.6%) |  |
| 3 | 9 (8.2%) | 20 (11.1%) | 7 (13.7%) |  |
| 4 | 12 (10.9%) | 28 (15.6%) | 2 (3.9%) |  |
| 5 | 5 (4.5%) | 17 (9.4%) | 10 (19.6%) |  |
| 6 | 4 (3.6%) | 14 (7.8%) | 5 (9.8%) |  |

The data are presented as the mean ± standard deviation, number (%), or median [interquartile range]. *Normoglycemia vs. overt hyperglycemia, p<0.05, Bonferroni post-hoc test. ^†^Normoglycemia vs. moderate hyperglycemia, p<0.05, Bonferroni post-hoc test. CAOD, coronary artery obstructive disease; SBP, systolic blood pressure; DBP, diastolic blood pressure; MBP, mean blood pressure; WBC, white blood cell; ESR, erythrocyte sedimentation rate; MRI, magnetic resonance imaging; SAH, subarachnoid hemorrhage; NIHSS, National Institutes of Health Stroke Scale; IQR, interquartile range; mRS, modified Rankin Scale.
